# Narcissism and self-esteem: A nomological network analysis

**DOI:** 10.1371/journal.pone.0201088

**Published:** 2018-08-01

**Authors:** Courtland S. Hyatt, Chelsea E. Sleep, Joanna Lamkin, Jessica L. Maples-Keller, Constantine Sedikides, W. Keith Campbell, Joshua D. Miller

**Affiliations:** 1 University of Georgia, Athens, Georgia, United States of America; 2 Michael E. DeBakey Veterans Affairs Medical Center, Baylor College of Medicine, Veterans Affairs South Central Mental Illness Research, Education and Clinical Center, Houston, Texas, United States of America; 3 Emory University School of Medicine, Atlanta, Georgia, United States of America; 4 University of Southampton, Southampton, United Kingdom; 5 Aalborg University, Aalborg, Denmark; Universitat Wien, AUSTRIA

## Abstract

Similarity between narcissism and self-esteem seems intuitive, as both capture positive perceptions of the self. In the current undertaking, we provide a broad comparison of the nomological networks of grandiose narcissism and explicit self-esteem. Pooling data from 11 existing samples (N = 4711), we compared the relations of narcissism and self-esteem to developmental experiences, individual differences, interpersonal functioning, and psychopathology. Both constructs are positively related to agentic traits and assertive interpersonal approaches, but differ in relation to agreeableness/communion. Self-esteem emerged as a wholly adaptive construct negatively associated with internalizing psychopathology and generally unrelated to externalizing behaviors. Unlike self-esteem, narcissism was related to callousness, grandiosity, entitlement, and demeaning attitudes towards others that likely partially explain narcissism’s links to maladaptive outcomes.

## Introduction

Narcissism is a personality construct typically characterized by grandiosity, vanity, entitlement, and exploitativeness. Since the publication of classic works by Freud [1], Kohut [2], and Kernberg [3], narcissism has been the subject of substantial interest in both the personality/social psychology literature (e.g., the Dark Triad; [4,5]) and clinical psychology/psychiatry literature [6,7]. In comparison, Rosenberg [8] defined self-esteem as a global, affective self-evaluation that can range from very negative to very positive. Considering this definition, the presence of a positive relation between narcissism and self-esteem seems intuitive, as both appear to capture positive perceptions of the self. Indeed, the data bear this out, as recent estimates suggest a small-to-medium effect for the relation between narcissism and self-esteem [9, 10]. It is easy to imagine how a prototypically grandiose narcissistic individual (i.e., arrogant, entitled, excessively self-promotional) might be construed as having particularly high self-esteem. However, existing empirical evidence suggests that framing narcissism as purely an exaggeration of high self-esteem may be an inaccurate depiction of these constructs. This is not to deny their apparent similarities, given that individuals with narcissistic traits, like those with high self-esteem, are likely to be perceived as confident and assertive, and to have pride in themselves [11]. Nevertheless, important differences between narcissism and self-esteem have been identified in terms of their developmental origins, trajectory over the lifespan, relations to prosocial and antisocial behavior, occupational performance, self-presentational tactics, and psychological health [12–19], which make it clear that self-esteem and narcissism are not isomorphic.

In the current article, we provide a relatively comprehensive comparison of the nomological networks of narcissism and self-esteem using an array of constructs relevant to various aspects of intra- and interpersonal psychological functioning that were assessed with diverse methodologies including self-reports, informant-reports, thin slice ratings, and laboratory paradigms. This work is drawn from three theoretical and empirical streams. The first is the agency model of narcissism, which posits that most of the self-concept positivity associated with narcissism is associated with agency but not communion, whereas the self-concept positivity associated with self-esteem is more evenly distributed across agency and communion [20]. The second stream shows that self-esteem is central to the psychological benefits of narcissism. That is, narcissism affords certain health benefits to the extent that it is associated with self-esteem; conversely, narcissism without self-esteem is positively linked to psychopathology [21]. The third stream is a new, integrative, trait-based model that grounds narcissism in three primary traits: agentic extraversion, (low) communion/agreeableness, and (low or high) neuroticism [22]. This model highlights the key areas to search for differences and similarities in narcissism and self-esteem.

Together, these theoretical and empirical streams lead to the following three hypotheses about the nomological networks [23] of narcissism and self-esteem. First, narcissism and self-esteem will be similarly related to traits associated with interpersonal agency. Second, narcissism and self-esteem will be differentially related to constructs associated with communion (e.g., aggression, antagonism, callousness), with narcissism positively related to antagonism-based traits (e.g., entitlement), behaviors (e.g., aggression), and disorders (e.g., psychopathy), and self-esteem negatively related to them. Third and finally, narcissism and self-esteem will be differentially related to traits (e.g., neuroticism), behaviors (e.g., laboratory aggression), and disorders (e.g., borderline) associated with functional maladaptivity, with narcissism showing null to moderate positive relations, and self-esteem demonstrating moderate to strong negative relations. In short, although narcissism will correlate with self-esteem, we propose that scales assessing both will manifest distinct nomological networks, which will showcase similarities in agency but differences in communion and degrees of adaptivity.

There are important nuances when considering these hypotheses. In line with the aforementioned third theoretical/empirical stream, the majority of predominant models of narcissism are not unidimensional, but rather they conceptualize narcissism as a multidimensional amalgam of several primary trait domains. Throughout the current manuscript, we generally refer to these traits in terms of agency and communion, but we note that several prominent models have different terms for iterations of these core constructs. For example, Five-Factor Model (FFM) conceptualizations of narcissism refer to the primary trait domains as extraversion and agreeableness [22]. Other conceptualizations of narcissism reflect the distinct intra-/interpersonal strategies that narcissistic individuals use to maintain their superior social standing, and domains; under this model [24], the domains agency and communion are reflected in the subscales admiration (i.e., wanting to be evaluated highly) and rivalry (i.e., wanting others to be evaluated poorly), respectively. Finally, the recently developed narcissism spectrum model [25] includes the central trait “entitlement,” as well as poles titled “grandiosity” and “vulnerability.” Grandiosity, which captures hubris and exhibitionism, is largely akin to agency/extraversion/admiration, whereas the central trait entitlement is akin to (low) communion/(low) agreeableness/rivalry.

In the current investigation, we are unable to speak directly to nomological networks of each of these trait terms. However, the NPI contains the subscales Leadership/Authority (LA), Grandiose Exhibitionism (GE), and Exploitativeness/Entitlement (EE), which can approximate agency and communion, the primary traits of interest. Prior work suggests that each of the NPI subscales is associated with low levels of communal traits (e.g., agreeableness), but that the EE subscale is the component most strongly associated with (low) communion [26]. On the other hand, the LA and GE subscales have been shown to largely capture agentic traits (i.e., extraversion, admiration, grandiosity [27]). Thus, in addition to the nomological network associated with the narcissism composite (i.e., NPI total score), the current analyses speak to these multidimensional models of narcissism by examining the differential criteria relations associated with the NPI subscales.

### Narcissism, self-esteem, and extraversion/agency vs. agreeableness/communion

Both self-esteem and narcissism are associated with feeling positively about the self, but not necessarily in the same way. One critical difference between the two appears to be the extent to which positive self-evaluative criteria are unlimited and available to all (self-esteem) or are finite and only attainable by those with ostensibly special abilities (narcissism). Campbell and colleagues [20] framed this distinction in terms of *communal* and *agentic* qualities of the self. Communal traits (e.g., warmth, nurturance, agreeableness) connect the self to the larger social world (and thus are plentiful), whereas agentic traits (e.g., surgency, action, skill) differentiate the self from others and thus are relatively scarce [24]. Put otherwise, communal traits allow one to “get along,” whereas agentic traits allow one to “get ahead” [28, 29].

Indeed, this is evident in the scales often used to assess these constructs. The vast majority of studies that assess self-esteem use the Rosenberg Self-Esteem Scale (RSES; [8]), given its psychometric properties and considerable cross-cultural support [30–32]. Items such as “I take a positive attitude toward myself” and “I feel that I have a number of good qualities,” gauge individuals’ cognitions and emotions about themselves. The RSES also includes items such as “I feel that I’m a person of worth, at least on an equal plane with others” and “I am able to do things as well as most other people.” Inherent in the interpretation of these items is Rosenberg’s [8] distinction between adequacy and superiority (p. 62). This measure reflects adequacy, and thus permits an individual with high self-esteem simultaneously to make positive evaluations about themselves and others. Individuals with high self-esteem do not necessarily see the world as a “zero-sum game,” in which some individuals will be characterized as winners and some as losers; that is, although they feel positively about themselves, this does not require that they denigrate others in comparison. The adequacy-reflecting RSES items stand in contrast to superiority-reflecting or “win-lose” items found in measures of narcissism such as the NPI [33], which includes “I think I am a special person,” “If I ruled the world, it would be a much better place,” “I like having authority over other people,” and “I find it easy to manipulate people.” Thinking positively about oneself does not necessitate preventing others from doing so, but manipulating others or wanting power over others implies putting one’s presumed competitors in a weaker or denigrated position.

Though the terms come from different scholarly traditions, agency and Big Five/FFM [34] extraversion are closely aligned (theoretically and psychometrically), as they both capture an active and assertive interpersonal approach characterized by social dominance [35, 36], although extraversion is broader than this and also captures more communal pieces too (e.g., warmth, gregariousness, experience of positive affect). Similarly, agreeableness and communion are both traits that involve tendencies toward social harmony and altruism. Narcissism, at least the grandiose dimension, is linked to low FFM agreeableness [37] and high agency/extraversion [38]. Furthermore, narcissistic individuals regard communal traits as less personally relevant than agentic traits [39, 40]. This combined high agency/extraversion paired with low communion/agreeableness profile (i.e., “disagreeable extraverts”; [41]) manifests itself in interpersonal skills (e.g., confidence) and strategies (e.g., self-promotion), as well as in intrapsychic mechanisms (e.g., self-serving bias; [42, 43]).

Self-esteem also bears strong relations with agency/extraversion. In a multinational survey of 106 countries [44], extraversion emerged as a universal correlate of self-esteem, but agreeableness did not, supporting the hypothesis that self-esteem, like narcissism, is associated with being assertive and active in one’s social world. Thus, one of the main differences lies in narcissism and self-esteem’s relations to the communal domain. Taken together, the parallels between narcissism and self-esteem lie in the realm of agency and extraversion, whereas their relations bifurcate on more communal and agreeable traits, such that narcissism is uniquely associated with low levels of communal traits and behaviors. We hypothesized that the NPI subscales will offer a more nuanced depiction of these relations. We expect that LA and GE will correlate most strongly with variables related to agency, and thus these subscales will have comparable relations to self-esteem for these traits. Alternately, we hypothesized that the EE subscale will show the strongest relations with variables related to low communion, underscoring that narcissism is best conceptualized as a construct that includes features of both high agency and low communion.

### Narcissism, self-esteem, and neuroticism/maladaptive functioning

A second critical difference concerns the degree of functional adaptivity associated with narcissism and self-esteem. In a large sample (N = 326,641; [45]), self-esteem was strongly related to emotional stability (i.e., low neuroticism; *r* = .50) and extraversion (*r* = .38). Given that emotional stability/neuroticism is the personality dimension most strongly associated with internalizing disorders [46], self-esteem may either act as a buffer against this form of psychopathology or result from good psychological health. Regardless, self-esteem’s relation to extraversion suggests that it is predominantly associated with a disposition toward positive emotions. This claim is corroborated by self-esteem’s negative relation to the onset of mood disorders as well as poorer physical health [47, 48]. Although narcissism is also related to emotional stability (*r* = .13; [49]), this relation is relatively small and much weaker than that found for self-esteem. As noted, Sedikides and colleagues [21] reported that the extent to which narcissism is associated with self-esteem explains any positive association between narcissism and (low) neuroticism. Thus, whereas narcissism appears to be somewhat negatively related to internalizing pathology, self-esteem appears to be a stronger protective factor.

Additionally, Robins and colleagues [45] found that self-esteem showed small, positive relations to conscientiousness (*r* = .24) and agreeableness (*r* = .13). Several meta-analyses suggest that these domains of personality are the most potently related to antisocial behavior (e.g., violence, substance use; [46, 50]). On the other hand, narcissism is characterized–both theoretically [51–53] and empirically [37, 54], by low agreeableness/high antagonism. Furthermore, narcissism is robustly linked to aggression, whereas self-esteem is typically unrelated to it [55–57]. Thus, whereas self-esteem appears to bear generally small and negative relations with externalizing behaviors, narcissism appears to be a key risk factor for externalizing pathology related to antagonism [58], particularly negative interpersonal behaviors that follow ego threat [59]. In sum, self-esteem seems to function as a protective factor against internalizing psychopathology, and to manifest small and negative relations to externalizing behavior. Alternately, whereas narcissism exhibits small and negative relations to internalizing psychopathology, it is a significant risk factor for externalizing behavior. We hypothesized that these differences will be reflected in the NPI subscale relations, such that the EE subscale bears the largest relations to indices of antagonism and externalizing behaviors. Additionally, considering the LA and GE subscales relations to the agentic traits, we hypothesized that these subscales will have the largest negative relations to internalizing psychopathology.

### What the current investigation is

We aim to provide a broad-scale, relatively comprehensive examination of the nomological networks of narcissism and self-esteem in order to determine where they converge and where they diverge, particularly with regard to agency/extraversion, communion/agreeableness, and neuroticism/adaptive functioning. Specifically, we compare explicit (as opposed to implicit) self-esteem and grandiose (as opposed to vulnerable) narcissism in terms of: (a) retrospective reports of developmental experiences, (b) individual differences in both general and pathological personality (e.g., FFM personality, DSM-5 personality disorders, and personality disorder traits), (c) approaches to interpersonal bonds (e.g., experiences in close relationships), and (d) relations with various putatively relevant outcomes including internalizing symptoms (e.g., depression) as well as externalizing behaviors (e.g., aggression). Although we derived many of our criteria from self-report inventories, we made use of a multitude of approaches, including informant-reports, social network analyses, thin slice-based ratings, and laboratory tasks.

Given evidence that narcissism in a multidimensional construct [22, 24, 25], as noted earlier, we examine both total narcissism scores from the NPI and its three subscales. Prior research suggests that the NPI LA and GE subscales measure primarily agentic traits (i.e., admiration, extraversion, or grandiosity in different models of narcissism), and to a lesser extent (low) communion, whereas the EE subscale primarily captures (low) agreeableness (i.e., rivalry, antagonism, and entitlement in different models of narcissism) consistent with the importance of these domains in grandiose narcissism) Thus, the current analyses can also speak to multidimensional models of narcissism that include these components.

Consistent with the extant literature base, we hypothesized that narcissism (operationalized as NPI total score) and self-esteem would evince similar correlations with constructs related to agency and extraversion, but would diverge in terms of constructs related to communion and agreeableness. Additionally, we expected that self-esteem (but not narcissism) would emerge as a relatively robust negative correlate of psychological distress—trait and state, as well as pathological intra- and interpersonal functioning. In terms of the NPI subscales, we hypothesized that the agentic aspects of narcissism will be reflected in the nomological network relations of the LA and GE subscales, such that the LA and GE subscales would demonstrate the largest positive relations to agentic traits and negative relations to internalizing psychopathology. Finally, we hypothesized that the EE subscale would bear the largest relations to the low communion and related externalizing behaviors.

### What the current investigation is not

The literatures on self-esteem and narcissism each include a relevant counterpart: implicit self-esteem and vulnerable narcissism. We have chosen not to focus on these variables for two reasons. First, implicit self-esteem, commonly defined as “uncontrollable, automatic feelings toward the self,” differs from explicit self-esteem or “deliberate, controllable feelings toward the self,” which is assessed by the RSES [60]. The “mask model of narcissism,” evident in the psychodynamic writings of Kernberg [61], emphasizes the role of implicit self-esteem, and proposes that narcissistic individuals develop and maintain overtly grandiose social presentation that acts as a buffer against covert feelings of inferiority rooted in interpersonal experiences from early in development. Common assessment strategies for implicit self-esteem include Implicit Associations Tests (IATs; [62]) and Name Letter Tests (NLTs; [63]). However, although explicit self-esteem is consistently correlated with narcissism, implicit measures of self-esteem are either uncorrelated with narcissism (IAT) or weakly correlated with narcissism in the unexpected (i.e., negative) direction (NLT), as suggested by a meta-analysis ([60]; but see [64]). Thus, we excluded implicit self-esteem due to both psychometric difficulties and a dearth of literature.

Second, vulnerable narcissism is characterized by shame, emotional reactivity, distrust, and hypersensitivity to criticism, along with self-absorption, entitlement, and low self-esteem [65, 66]; in fact, trait neuroticism serves as an excellent proxy for vulnerable narcissism [67]. Narcissistic grandiosity and vulnerability share a common core of low agreeableness, but diverge strongly in relation to other personality constructs, particularly neuroticism and extraversion [22]. Despite this convergence on agreeableness, these profiles are essentially unrelated, or strongly negatively related, if their shared variance is removed [65, 67]. In addition to the empirical distinction, academics, clinicians, and lay-persons alike describe the prototypically narcissistic individual in terms consistent with grandiose narcissism [68]. As such, we eschew vulnerable narcissism and focus on its empirically defined and consensually supported counterpart.

## Methods

### Participants

All studies were approved by the University of Georgia IRB, and all participants provided informed consent. Sample 1 consisted of 238 undergraduate students (60% women; M_age_ = 19.1 years, SD_age_ = 1.3 years; 83% Caucasian) recruited from the research participant pool at a large southeastern university. Data from this sample have been previously published [49].

Sample 2 consisted of 1,056 adults, who were recruited through Amazon’s Mechanical Turk (MTurk) website. Of the 1,056 participants, 140 were excluded for missing more than 20% of the data or for invalid responding. The resulting data set included 916 adults (62% women; M_age_ = 34.4 years, SD_age_ = 13.0 years; 84% Caucasian, 5.3% African American, 4.5% Multiracial, 4.1% Asian). Data from this sample were previously published [69].

Sample 3 consisted of 277 adults (65% women; M_age_ = 31.3 years, SD_age_ = 11.0 years; 85% Caucasian), who were recruited through Amazon’s MTurk. Data from this sample were previously published [66].

Sample 4 consisted of 306 adults (43% women; M_age_ = 29.7 years, SD_age_ = 10.2 years; 49% Asian, 46%; Caucasian), who were recruited via Amazon’s MTurk. Data from this sample were previously published [70].

Sample 5 consisted of 148 undergraduate students (53% women; M_age_ = 19.2 years, SD_age_ = 1.5 years; 86% Caucasian) recruited from the research participant pool at a large southeastern university. Data from this sample were previously published [71].

Sample 6 consisted of 361 undergraduate students (62% women; M_age_ = 19.1 years, SD_age_ = 1.7 years; 87% Caucasian) recruited from the research participant pool at a large southeastern university. Additionally, to obtain informant report, a packet of questionnaires was sent to the home of all participants’ parents. Data from this sample were previously published [65].

Sample 7 consisted of 274 undergraduate students (67% women; M_age_ = 19.4 years, SD_age_ = 1.7 years; 77% Caucasian, 12% African American, 8% Asian) recruited from the research participant pool at a large southeastern university. Data from this sample were previously published [72].

Sample 8 consisted of 271 undergraduate students (56% women; M_age_ = 19.3 years, SD_age_ = 1.26 years; 86% Caucasian) recruited from the research participant pool at a large southeastern university. Data from this sample were previously published [73].

Sample 9 consisted of 993 undergraduate students (60% women; M_age_ = 19.3 years, SD_age_ = 1.6 years; 81% Caucasian) recruited from the research participant pool at a large southeastern university. Informant reports were collected as well (68.59% of informants identified as a friend of the target participant, 12.84% identified as a sibling, 5.29% identified as a romantic partner, 4.7% identified as a roommate, and 3.6% identified as a nonsibling family member). Data from this sample were previously published [74].

Sample 10 consisted of 865 adults, who were recruited from MTurk. Participants were excluded for invalid responding, for finishing the study in a time deemed invalid (≤ 20 mins), and for failing to respond to 25% or more of the items (N = 262). The final sample consisted of 603 individuals (63% women; M_age_ = 37.0 years, SD_age_ = 11.8 years; 79% Caucasian). Data from this sample were previously published [68].

Sample 11 consisted of 230 undergraduate students (54.8% women; *M*_age_ = 19.3 years, *SD*_age_ = 1.5 years; 68% Caucasian, 13% African American, 13% Asian-American, 4% Hispanic or Latino) recruited from the research participant pool at a large southeastern university. Due to reported doubt about the laboratory aggression paradigm expressed during the manipulation check, 10 participants were excluded, leaving a final sample of 220. The full procedure and data from this sample were previously published [75].

### Materials and analyses

#### Narcissistic personality inventory

The Narcissistic Personality Inventory (NPI; [33]) is a 40-item, forced-choice, self-report measure of trait narcissism (αs ranged from .77 in *Sample 10* to .90 in *Sample 3*) with subscales that measure Leadership/Authority (LA; αs ranged from .73 in *Sample 4* to .82 in *Sample 3*), Grandiose Exhibitionism (GE; αs ranged from .72 in *Sample 11* to .79 in *Sample 2* and *3*), and Entitlement/Exploitativeness (EE; αs ranged from .41 in *Sample 7* to .62 in *Sample 3*). In *Sample 10*, we used a Likert (1–5; [76]) version of the NPI-40: (Total: *α =* .*77)*, LA (*α* = .90), GE (*α* = .82), and EE (*α* = .68). In *Sample 9*, we used the NPI-16 [77], which generates a global narcissism score only (α = .71). In *Sample 11*, we used the NPI-13 [27], which similarly generates a total score (α = .70) and contains 3 subscales: LA (α = .69), GE (α = .61), and EE (α = .52).

#### Rosenberg self-esteem scale

The Rosenberg Self-Esteem Scale (RSES; [8]) is a 10-item global measure of self-esteem. Alphas ranged from .75 (*Sample 7*) to .92 (*Sample 10* and *11*).

#### Parenting warmth and monitoring scale

The Parental Warmth and Monitoring Scale (PWMS; [78]) is a 24-item, self-report measure of the degree of warmth (*Sample 2*, *6*, and *8* αs = .88, .82, and .72) and parental supervision (*Sample 2*, *6*, and *8* αs = .87, .80, and .76) individuals received as adolescents.

#### Psychological control scale

The Psychological Control Scale (PCS; [79]) is a 16-item, self-report measure of the level of psychological control (*Sample 2*, *6*, and *8* αs = .88, .85, and .81) asserted by one’s parents. We used the mean of the mother and father ratings, if both were provided. We used only one parent rating, if only that was available.

#### Experiences in close relationships—Revised

The Experiences in Close Relationships–Revised (ECR-R; [80]) is a 36-item, self-report measure of two adult attachment styles: Avoidance (*Sample 1*, *2*, and *6* αs = .93, .96, and .93) and Anxiety (*Sample 1*, *2*, and *6* αs = .93, .95, and .93).

#### Child abuse and trauma scale

The Child Abuse and Trauma Scale (CATS; [81]) is a 38-item, self-report measure of physical, verbal, emotional, and sexual abuse. We used only the items included in the revised scales described by Poythress and colleagues [82] that yield a total score (*Sample 2* and *6* αs = .94 and .93) along with subscales for physical (*Sample 2* and *6* αs = .90 and .71), verbal (*Sample 2* and *6* αs = .87 and .77), emotional (*Sample 2* and *6* αs = .91 and .82), and sexual (*Sample 2* and *6* αs = .83 and .86) abuse.

#### Five-factor model measures

We used the Revised NEO Personality Inventory (NEO PI-R; [34]) and the IPIP NEO PI-R (IPIP NEO-120; [83]). The NEO PI-R is a 240-item measure and the IPIP NEO PI-R is a 120-item measure of the FFM. The higher order domains of the FFM include Neuroticism (αs ranged from .89 in *Sample 2* to .94 in *Sample 4* and *Sample 11*), Extraversion (αs ranged from .86 in *Sample 2* to .91 in *Samples 4*, *8*, and *11*), Openness (αs ranged from .72 in *Sample 2* to .89 in *Samples 1* and *5*), Agreeableness (αs ranged from .69 in *Sample 2* to .92 in *Sample 5*), and Conscientiousness (αs ranged from .83 in *Sample 2* to .92 in *Samples 4*, *5*, *6*, and *8*). Each of the FFM domains is underlain by six more specific facets. In *Sample 7*, we used the Big Five Inventory [84]. Alpha coefficients ranged from .78 (Agreeableness) to .85 (Extraversion). In *Sample 9*, we used the Ten Item Personality Measure (TIPI; [83]). Alphas ranged from .36 (Openness) to .65 (Extraversion).

#### Informant measures of five factor model traits

For our informant measure, we used the TIPI [85], a 10-item measure of the five factor dimensions of personality, with two items per each of the factors. In *Sample 9*, alpha for domains ranged from .42 (Openness) to .68 (Extraversion). In *Sample 6*, parents of participants completed an informant version of the NEO Five-Factor Inventory [34], which has 60 items for assessing the five broad domains. Alphas for the informant-reported FFM domains ranged from .63 (Openness) to .90 (Conscientiousness).

#### Thin slices

Following the protocol described by Oltmanns and colleagues [86], participants in *Sample 1* was individually videotaped while answering the following question for 60 seconds: “What do you enjoy doing?” Each video clip was then rated by, on average, 11 raters who were doctoral students in a clinical psychology program. We calculated interrater reliability using intraclass correlations, which ranged from .77 (likeability) to .92 (physical attractiveness) with a median of .86. We created composites for subsequent analyses by taking the mean of all available ratings.

#### Behavioral inhibition/activation scale

The Behavioral Inhibition/Activation Scale (BIS/BAS; [87]) is a 24-item self-report measure of behavioral inhibition (*Sample 6* α = .79) and activation, which is comprised of three subscales: Reward Responsiveness (*Sample 6* α = .85), Drive (*Sample 6* α = .82), and Fun Seeking (*Sample 6* α = .80).

#### Social vignettes

In *Sample 1*, participants read 12 vignettes [88] describing a hypothetical interaction in which another person performs a behavior that might be considered provocative to the participant (e.g., “You are at a local dance club. While you are dancing a stranger bumps into you very roughly”); four were “hostile,” four were “ambiguous,” and four were “unintentional.” The participants were then asked questions (1 = *not at all likely*, 11 = *extremely likely*) assessing the likelihood of experiencing anger during the interaction (α = .87), expressing anger towards the other individual in the interaction (α = .88), being rude (α = .88), yelling or swearing (α = .88), threatening the other person if the situation was not resolved (α = .89), and using physical force if the situation was not resolved (α = .87). We summed the answers for each of these six responses across the 12 vignettes.

#### Resource dilemma task

This task is based on the “tragedy of the commons” dilemma [89]. In *Sample 1*, participants were asked to believe they owned a timber company and were competing with three similar companies to harvest trees in the same national forest. Three dependent variables were created from this task: acquisitiveness, apprehensiveness, and harvest bids. The dilemma in this situation is that, if all four companies put their own profit motives first and harvest too much, the forest will be de-forested, leaving no available resources for all four companies. We measured acquisitiveness and apprehensiveness each with one question. We measured the harvest bids variable (α = .84) with five questions (one bid per year).

#### Interpersonal adjective scale

The Interpersonal Adjective Scale (IAS; [90]) contains 64 adjectives, scored on a 1 to 8 scale, that provide scores on the interpersonal circumplex. The scale includes eight octant scores and scores on the two primary axes of dominance and nurturance. In *Sample 3*, the alphas for the octants ranged from .79 (Unassuming-Ingenuous) to .91 (Cold-hearted), with a median of .87.

#### Inventory of interpersonal problems

The Inventory of Interpersonal Problems (IIP; [91]) is a 127-item, self-report measure of problems associated with interpersonal behaviors and associated distress. In *Sample 3*, alphas for the octants ranged from .82 (Intrusive) to .90 (Nonassertive).

#### Social network report

In *Sample 5*, participants were asked to list 30 individuals (alters) perceived as most important to them within the past year. Participants rated each alter’s gender, race, student status if applicable (e.g., freshman), origin of relationship (school, family, or other), relationship characteristics, and length of relationship. Participants also rated the personality of each alter using the TIPI as well as relationship characteristics. They also used additional items to rate each alter on self-centeredness, attractiveness, social status, leadership, narcissism, intelligence, likability, and kindness. Finally, they rated perceptions of relationship characteristics for frequency of arguing with each alter, frequency of envying each alter, frequency of comparing self to each alter, perceived familiarity with each alter, and perceived closeness to each alter on the same.

#### Personality assessment inventory

The Personality Assessment Inventory (PAI; [92]) is a self-report instrument that uses 344 items to assess psychopathological constructs such as depression, anxiety, treatment rejection, and antisocial and borderline personality disorders, among others. In *Sample 7*, alphas ranged from .69 (Treatment Rejection) to .91 (Anxiety, Depression), with a median of .88.

#### Brief symptom inventory

The Brief Symptom Inventory (BSI; [93]) is a 53-item measure of psychological symptoms experienced during the past week that includes symptom scales and a global severity index (GSI). We used only the GSI. Alphas ranged from .96 (*Samples 5* and *8*) to .97 (*Samples 1* and *6*).

#### Patient-reported outcomes measurement information system

The Patient-reported Outcomes Measurement Information System (PROMIS; [94]) consists of brief questionnaires designed to assess the experience of anxiety (*Samples 3*, *9*, and *10* αs = .94, .92, and .96) and depression (*Samples 3*, *9*, and *10* αs = .96, .95, and .97) over the past seven days.

#### Positive and negative affect schedule

The Positive and Negative Affect Schedule (PANAS-X; [95]) is a 60-item measure of positive (*Samples 1*, *2*, and *6* αs = .84, .89, and .87) and negative affect (*Samples 1*, *2*, and *6* αs = .85, .92, and .83).

#### Crime and analogous behavior scale

The Crime and Analogous Behavior scale (CAB; [96]) is a 25-item measure of various externalizing behaviors including substance use (αs ranged from .57 in *Sample 11* to .75 in *Sample 6*) and antisocial behavior (αs ranged from .47 in *Sample 11* to .68 in *Sample 2*).

#### Reactive-proactive aggression questionnaire

The Reactive Proactive Aggression Questionnaire (RPAQ; [97]) consists of 23 self-report items assessing total aggression (*Sample 10* and *11* αs = .87 and .84) and two aggression scales: proactive aggression (*Sample 10* and *11* αs = .82 and .75 and reactive aggression (*Sample 10* and *11* αs = .82 and .79).

#### Response-choice aggression paradigm

The Response-Choice Aggression Paradigm (RCAP; [98]) is a laboratory task used to quantify aggressive behavior. For half of the participants, the RCAP was presented as a reaction time (RT) competition, wherein participants compete in 30 RT trials against an ostensible “opponent” who is alleged to be sitting next door. A computer program predetermined the outcomes such that each participant had an identical schedule of 15 “wins” and 15 “losses.” After each trial, regardless of outcome, participants had an opportunity to administer a shock to their partner, but they did not have to do so. In the current sample, half of the participants completed a “no-competition” form of this paradigm, wherein they did not receive feedback about “wins/losses”, and their counterpart was not depicted as an “opponent”. Intensity of shock ranged from 1 (lowest) to 10 (highest). We computed a composite aggression variable by summing the Z scores of each aggression index (shock frequency, mean shock intensity, and mean duration).

#### Psychological entitlement scale

The Psychological Entitlement Scale (PES; [99]) is a 9-item self-report measure of the extent to which individuals believe that they deserve and are entitled to more than others (αs ranged from .86 in *Sample 1* to .91 in *Samples 2* and *5*]).

#### Personality inventory for DSM-5

The Personality Inventory for DSM-5 (PID-5; [100]) is a self-report inventory developed to assess traits included in the DSM-5 alternative dimensional model. The inventory contains 220 items and is aggregated to yield scores for the 25 individual traits and the five broad domains. In *Sample 4*, alphas ranged from .68 to .94 for the facets, with a median of .86.

#### Personality diagnostic questionnaire-4+

The Personality Diagnostic Questionnaire-4+ (PDQ-4+; [101]) is a 99-item self-report measure of DSM-IV PDs. PD symptom counts are computed by summing the items endorsed for each PD. In *Sample 8*, alphas for symptom counts ranged from .23 (OCPD) to .64 (PPD), with a median of .52.

#### Structured clinical interview for DSM-IV personality disorders—Personality questionnaire

The Structured Clinical Interview for DSM-IV Personality Disorders—Personality Questionnaire (SCID-II P/Q; [102]) is a 119-item self-report questionnaire designed to assess the DSM-IV PDs. Alphas ranged from .44 (i.e., Obsessive Compulsive in *Sample 1*) to .92 (i.e., Antisocial in *Sample 9*) with a median of .73.

#### Elemental psychopathy assessment

The Elemental Psychopathy Assessment (EPA; [103]) is a 178-item self-report measure of psychopathy that provides a total score as well as scores on 18 subscales measuring psychopathy. We used only the total score for analyses (*Sample 1* α: = .95).

#### Levenson’s self-report psychopathy scale

The Levenson’s Self-report Psychopathy Scale (LSRP; [104]) is a 26-item self-report inventory designed to measure psychopathy. We used only the total score (*Sample 6* and *8* αs: = .85 and .83)

#### Self-report psychopathy scale: Version III

The Self-Report Psychopathy Scale: Version III (SRP-III; [105]) is a 64-item measure of psychopathy with subscales of Interpersonal Manipulation, Callous Affect, Erratic Lifestyle, and Antisocial Behavior. We used only the total score (*Samples 6* and *9* αs = .93 and .94).

#### Short dark triad-3

The Short Dark Triad-3 (SD3; [106]) is a 27-item measure of narcissism, Machiavellianism (*Sample 2* α = .81), and psychopathy (*Sample 2* α = .79). We used only the latter two constructs.

#### Narcissistic grandiosity scale

The Narcissistic Grandiosity Scale (NGS; [107]) is a measure of grandiose narcissism, which requires participants to rate themselves on 16 adjectives such as “superior” and “omnipotent” (1 = *not at all*, 7 = *extremely*). Alphas ranged from .95 (*Sample 5*) to .96 (*Sample 3*, *4*, and *7*).

#### Hypersensitive narcissism scale

The Hypersensitive Narcissism Scale (HSNS; [108]) is a 10-item self-report measure that reflects hypersensitivity, vulnerability, and entitlement. Alphas ranged from .67 (*Sample 5* and *6*) to .81 (*Sample 3*).

#### MACH-IV

The MACH-IV [109] is a 20-item measure of the personality trait of Machiavellianism (*Sample 9* α = .72).

#### Nomological network analyses

To assess the empirical networks that characterize narcissism and self-esteem, our primary procedure was to compare the associations of narcissism to the various criteria variables with the associations for self-esteem. When only one sample with a given criteria variable was available, we used simple bivariate correlations. When multiple samples were available, we used meta-analyses (see description below). We could then compare the correlations individually (e.g., with z-tests) as well as test the absolute similarity of the overall patterns of correlations via intraclass correlations (see description below).

#### Meta-analyses

Due to the large number of overlapping measures in our samples, we conducted “mini” meta-analyses using the reported samples whenever possible. We used random effects models to estimate the mean Pearson’s *r* between narcissism (and NPI subscales), self-esteem, and the variables reported above [110]. In line with common meta-analytic practice, we standardized all *r* values using a Fisher’s z transformation prior to aggregation, then back transformed when presented in final form [111]. We used the MeanES macro for SPSS version 24.0 to calculate the aggregated effect [112]. Then, we used a version of Steiger’s z-test to determine if a variable’s correlations with narcissism and self-esteem were significantly different [113].

#### Intraclass correlation analyses

To compare the total profile similarity for narcissism and self-esteem, double-entry Q-correlations were used as they assess the absolute similarities of these profiles (rather than their relative similarity; [114]).

## Results

### The relation between narcissism and self-esteem

First, we meta-analytically computed the relation between narcissism and self-esteem across all of the current samples (k = 11, N = 4711). Results of the random-effects estimate the relation at *r* = .28 (95% Confidence Interval = .21 to .35). Across samples, the strength of the relations varied from *r* = .10 (N = 306) to *r* = .43 (N = 270), with a median of *r* = .30. In term of the NPI subscales (k = 10, N = 3718), the LA subscale displayed a meta-analytic relation with self-esteem that was medium in magnitude (*r* = .32; 95% Confidence Interval = .24 to .37). The GE subscale displayed a small meta-analytic relation (*r* = .23; 95% Confidence Interval = .16 to .29), while the EE subscale displayed a null meta-analytic relation (*r* = .01; 95% Confidence Interval = -.08 to .11).

### Nomological network criteria

#### Developmental experiences

We present results of these analyses in Table 1. Broadly, the narcissism total score was generally unrelated to self-reported adverse developmental experiences, whereas self-esteem evinced negative relations with parental intrusiveness, abuse, and anxious and avoidant attachment styles. Additionally, self-esteem was positively related to parental warmth and monitoring, whereas narcissism was unrelated to these variables. Narcissism and self-esteem differed significantly on 10/10 (100%) of the developmental variables assessed.

**Table 1:** Developmental experiences.

	Narcissism	Self-Esteem	NPI-LA	NPI-GE	NPI-EE
***Parenting*** (k = 3, N = 1688)	*r*	*r*	*r*	*r*	*r*
Parent Warmth	.00^a^ (-.04 –.05)	.26^b^ (.19 –.35)	.01 (-.03 –.06)	.03 (.02 –.08)	-.10 (-.16 –-.05)
Parent Monitoring	-.07^a^ (-.12 –-.02)	.16^b^ (.11 –.20)	-.06 (-.10 –-.01)	-.03 (-.08 –.02)	-.11 (-.16 –-.07)
Parent Intrusive	.09^a^ (.04–14)	-.28^b^ (-.33 –-.24)	.04 (-.06 –.10)	.07 (.03 –.12)	.12 (.07 –.17)
***Attachment*** (k = 3, N = 1655)					
Anxious	-.11^a^ (-.16 –-.05)	**-.42**^b^ (-.50 –-.34)	-.13 (-.21 –-.06)	-.06 (-.14 –.00)	.16 (.12 –.21)
Avoidant	-.06^a^ (-.11 –-.01)	**-.43**^b^ (-.54 –-.29)	-.08 (-.12 –-.03)	-.11 (-.16 –-.06)	.17 (.12 –.22)
***Abuse*** (k = 2, N = 1417)					
Abuse Total	.04^a^ (-.02 –.10)	**-.35**^b^ (-.49 –-.19)	.02 (-.08 –.12)	.00 (-.07 –.07)	.15 (.10 –.20)
Physical Abuse	.06^a^ (-.05 –.16)	-.26^b^ (-.37 –-.18)	.05 (-.05 –.15)	.00 (-.12 –.13)	.16 (.11 –.22)
Verbal Abuse	.03^a^ (-.02 –.08)	-.27^b^ (-.41 –-.13)	.03 (-.02 –.08)	.02 (-.03 –.08)	.09 (.04 –.14)
Sexual Abuse	.10^a^ (.05 –.15)	-.24^b^ (-.32 –-.16)	.09 (.04 –.14)	.07 (.00 –.13)	.18 (.13 –.23)
Emotional Abuse	-.02^a^ (-.07 –-.03)	**-.36**^b^ (-.50 –-.21)	-.04 (-.14 –.07)	-.04 (-.09 –.01)	.09 (.03 –.14)

*Note*. Bold = Effect sizes ≥.30; () = 95% Confidence Interval; LA = Leadership/Authority subscale of the NPI; GE = Grandiose/Exhibitionism subscale of the NPI; EE = Entitlement/Exploitativeness subscale of the NPI

^ab^superscripts are used to indicate that Steiger’s z-tests suggest these correlations are significantly different from one another.

The NPI LA and GE subscales were unrelated to parental warmth, monitoring, intrusiveness, and history of abuse (Table 1). LA and GE showed small, negative relations to anxious and avoidant attachment styles, whereas Entitlement/Exploitativeness (EE) showed small, positive relations to these attachment styles, as well as abuse. NPI EE was negatively correlated with warmth and monitoring, and positively correlated intrusiveness, though each of these relations were small in nature.

#### Personality traits

As hypothesized, total narcissism and self-esteem were strongly related to extraversion, as assessed by self-report, informant-report, and thin slice ratings (Table 2). Self-report, informant-report, and thin slice ratings suggest that narcissism is negatively related to agreeableness, whereas the relation between self-esteem and agreeableness is small but positive. Additionally, though the direction of the relations were the same, self-esteem was more strongly negatively related to neuroticism. Self-esteem was also generally much more strongly linked to conscientiousness than narcissism.

**Table 2:** Traits.

	Narcissism	Self-Esteem	NPI-LA	NPI-GE	NPI-EE
***FFM***	*r*	*r*	*r*	*r*	*r*
*Self* (k = 9, N = 2464)					
Neuroticism	-.19^a^ (-.19 –-.13)	**-.65**^b^ (-.71 –-.58)	-.27 (-.32 –-.22)	-.10 (-.13 –-.06)	.12 (.07 –.17)
Extraversion	**.42** (.35 –.48)	**.39** (.33 –.44)	**.44** (.37 –.51)	**.41** (.35 –.45)	.01 (-.10 –.09)
Openness	.04 (-.02 –.11)	.06 (-.01 –.14)	.02 (-.04 –.09)	.07 (.00 –.15)	-.09 (-.17 –-.02)
Agreeableness	**-.42**^a^ (-.49 –-.36)	.11^b^ (.06 –.17)	**-.35** (-.41 –-.28)	**-**.29 (-.33 –-.26)	**-.51** (-.55 –-.46)
Conscientiousness	.10^a^ (.04 –.15)	**.43**^b^ (.38 –.49)	.20 (.17 –.24)	-.03 (-.06 –.00)	-.10 (-.15 –-.05)
*Informant* (k = 2, N = 1325)					
Neuroticism	-.09^a^ (-.18 –.01)	-.30^b^ (-.45 –-.14)	-.19 (-.23 –-.09)	-.11 (-.22 –.01)	-.07 (-.18 –.03)
Extraversion	.23^a^ (.08 –.35)	.16^b^ (.11 –.22)	.11 (-.01 –.22)	.19 (.07 –.28)	-.17 (-.26 –-.06)
Openness	.03 (-.02 –.09)	.06 (.00 –.11)	.02 (-.10 –.14)	.10 (.00 –.21)	.17 (.07 –.26)
Agreeableness	-.22^a^ (-.26 –-.17)	.05^b^ (-.01 –.10)	-.17 (-.26 –-.07)	-.15 (-.24 –-.05)	-.12 (-.23 –-.02)
Conscientiousness	-.12^a^ (-.17 –-.07)	.13^b^ (.08 –.18)	-.01 (-.12 –.09)	-.09 (-.19 –.01)	-.02 (-.12 –.09)
*Thin Slice* (k = 1, N = 238)					
Neuroticism	-.29 (-.40 –-.17)	-.25 (-.37 –-.13)	**-.30** (-.41 –-.18)	-.29 (-.40 –-.17)	-.05 (-.18 –.08)
Extraversion	**.40** (.29 –.50)	**.30** (.18 –.41)	**.40** (.29 –.50)	**.42** (.31 –.52)	.12 (-.01 –.24)
Openness	.12 (-.01 –.24)	.03 (-.10 –.16)	.10 (-.03 –.22)	.08 (-.05 –.21)	.10 (-.03 –.22)
Agreeableness	-.09^a^ (-.21 –.04)	.13^b^ (.00 –.25)	-.12 (-.24 –.01)	.05 (-.08 –.18)	-.12 (-.24 –.01)
Conscientiousness	-.16 (-.28 –-.03)	-.07 (-.20 –.06)	-.18 (-.30 –-.05)	-.14 (-.26 –-.01)	-.08 (-.21 –.05)
Attractiveness	.23 (.11 –.35)	.12 (-.01 –.24)	.15 (.02 –.27)	**.30** (.18 –.41)	.12 (-.01 –.24)
Likeability	.20 (.07 –.32)	.24 (.12 –.36)	.15 (.02 –.27)	.24 (.12 –.36)	.02 (-.11 –.15)
Narcissism	**.35**^a^ (.23 –.46)	.05^b^ (-.08 –.18)	**.38** (.27 –.48)	**.30** (.18 –.41)	.13 (.00 –.25)
***BIS/BAS*** (k = 1, N = 361)					
Behavior Inhibition	**-.30** (-.39 –-.20)	**-.33** (-.42 –-.23)	**-.34** (-.43 –-.25)	-.07 (-.17 –.03)	-.09 (-.19 –.01)
Reward Responsiveness	.09^a^ (-.01 –.19)	.24^b^ (.14 –.33)	.03 (-.07 –.13)	.21 (.11 –.31)	-.10 (-.20 –.00)
Drive	**.42**^a^ (.33 –.50)	.22^b^ (.12 –.32)	**.38** (.29 –.46)	**.34** (.25 –.43)	.17 (.07 –.27)
Fun Seeking	.22 (.12 –.32)	.11 (.01 –.21)	.16 (.06 –.26)	.20 (.10 –.30)	.03 (-.07 –.13)

*Note*. Bold = Effect sizes ≥.30; () = 95% Confidence Interval; LA = Leadership/Authority subscale of the NPI; GE = Grandiose/Exhibitionism subscale of the NPI; EE = Entitlement/Exploitativeness subscale of the NPI

^ab^superscripts are used to indicate that Steiger’s z-tests suggest these correlations are significantly different from one another.

Narcissism and self-esteem were both positively related to behavioral activation variables (e.g., drive) and negatively related to behavioral inhibition. Furthermore, both narcissism and self-esteem were positively related to positive affect, but self-esteem was uniquely associated with low negative affect. Narcissism and self-esteem significantly differed on 15/24 (63%) of the trait variables assessed.

At the subscale level, the NPI subscales manifested moderate to large, negative correlations with the self-report FFM agreeableness, as well as small, negative correlations with the informant report agreeableness (Table 2). NPI LA and GE both bore strong, positive relations to self-report extraversion, thin slice rated extraversion and narcissism, and drive, whereas NPI EE was generally unrelated to extraversion.

#### Interpersonal functioning

In terms of social responding, total narcissism was uniquely associated with experiencing and expressing anger, as well directly confrontational responses such as yelling, threatening, and implementing physical aggression (Table 3). Self-esteem was weakly negatively related to these responses. Additionally, narcissism was positively related to a drive for acquisition in a resource-sharing dilemma, as well as bids for disproportionate resources, whereas self-esteem was unrelated to these variables.

**Table 3:** Interpersonal functioning.

	Narcissism	Self-Esteem	NPI-LA	NPI-GE	NPI-EE
***Social Responding*** (k = 1, N = 238) *r*		*r*	*r*	*r*	*r*
Experience anger	.17^a^ (.04 –.29)	-.20^b^ (-.32 –-.07)	.12 (-.01 –.24)	.15 (.02 –.27)	.24 (.12 –.36)
Express anger	**.37**^a^ (.25 –.47)	-.05^b^ (-.18 –.08)	**.31** (.19 –.42)	.26 (.14 –.37)	**.33** (.21 –.44)
Be rude	**.36**^a^ (.24 –.47)	-.06^b^ (-.19 –.07)	**.32** (.20 –.43)	.17 (.04 –.29)	**.38** (.27 –.48)
Yell	**.34**^a^ (.22 –.45)	-.09^b^ (-.21 –.04)	**.32** (.20 –.43)	.11 (-02 –.23)	**.36** (.24 –.47)
Threaten	**.32**^a^ (.20 –.43)	-.05^b^ (-.18 –.08)	**.31** (.19 –.42)	.12 (-.01 –.24)	**.36** (.24–47)
Use physical aggression	.23^a^ (.11 –.35)	-.03^b^ (-.16 –.10)	.26 (.14 –.37)	.07 (-.06 –.20)	.24 (.12 –.36)
***Negotiation*** (k = 1, N = 238)					
Acquisitiveness	.22^a^ (.10 –.34)	.00^b^ (-.13 –.13)	.22 (.10 –.34)	.17 (.04 –.29)	.19 (.06 –.31)
Apprehensiveness	-.06 (-.19 –.07)	-.15 (-.27 –-02)	-.03 (-.16 –.10)	-.09 (-.21 –.04)	.09 (-.04 –.21)
Harvest bids	.22^a^ (.10 –.34)	-.01^b^ (-.14 –.12)	.21 (.09 –.33)	.13 (.00 –.25)	.25 (.13 –.37)
***Interpersonal Adjectives*** (k = 1, N = 253)					
Love	.02^a^ (-.10 –.14)	.26^b^ (.14 –.37)	.05 (-.07 –.17)	.09 (-.03 –.21)	**-.34** (-.43 –-.21)
Dominance	**.53** (.42 –.61)	**.58** (.49 –.66)	**.56** (.47 –.64)	**.38** (.27 –.48)	.04 (-.08 –.16)
Assured-Dominant	**.53** (.44 –.61)	.**43** (.32 –.53)	**.58** (.49 –.66)	**.31** (.19 –.42)	.20 (.08 –.32)
Arrogant-Calculating	**.53**^a^ (.44 –.61)	.12^b^ (.00 –.24)	**.44** (.33 –.53)	**.36** (.25 –.46)	**.32** (.20 –.43)
Cold-Hearted	**.42**^a^ (.31 –.52)	-.17 ^b^ (-.29 –-.05)	**.30** (.18 –.41)	.27 (.15 –.38)	**.51** (.41 –.60)
Aloof-Introverted	-.10^a^ (-.22 –.02)	**-.51**^b^ (-.60 –-.41)	-.17 (-.29 –-.05)	-.13 (-.25 –-.01)	.29 (.17 –.40)
Unassured-Submissive	**-.38** (-.48 –-.27)	**-.49** (-.58 –-.39)	**-.42** (-.52 –-.31)	-.28 (-.39 –-.16)	.03 (-.09 –.15)
Unassuming-Ingenuous	**-.34** (-.44 –.23)	-.29 (-.40 –-.17)	**-.33** (-.44 –-.22)	-.22 (-.33 –-.10)	-.11 (-.23 –.01)
Warm-Agreeable	**-.30**^a^ (-.41 –-.18)	.13^b^ (.01 –.25)	-.19 (-.31 –-.07)	-.18 (-.30 –-.06)	**-.44** (-.53 –-.33)
Gregarious-Extraverted	.28^a^ (.16 –.39)	**.53**^b^ (.44 –.61)	**.32** (.20 –.43)	.27 (.15 –.38)	-.18 (-.30 –-.06)
***Interpersonal Problems*** (k = 1, N = 253)					
Domineering	**.45**^a^ (.35 –.54)	.22^b^ (.10 –.33)	**.43** (.32 –.53)	.27 (.15 –.38)	.26 (.14 –.37)
Vindictive	**.33**^a^ (.22 –.44)	.11^b^ (-.01 –.23)	**.36** (.25 –.46)	.12 (.00 –.24)	**.31** (.19 –.42)
Cold	.21 (.09 –.32)	.03 (-.09 –.15)	.20 (.08 –.32)	.09 (-.03 –.21)	.27 (.15 –.38)
Socially-Avoidant	**-.39** (-.49 –-.28)	-.28 (-.39 –-.16)	**-.35** (-.45 –-.24)	**-.34** (-.44 –-.23)	-.08 (-.20 –.04)
Unassertive	**-.42**^a^ (-.52 –-.31)	-.19^b^ (-.31 –-.07)	**-.44** (-.53 –-.33)	-.24 (-.35 –-.12)	-.15 (-.27 –-.03)
Exploitable	**-.30**^a^ (-.41 –-.18)	-.04^b^ (-.16 –.08)	**-.30** (-.41 –-.18)	-.15 (-.27 –-.03)	-.27 (-.38 –-.15)
Overly-Nurturant	-.12 (-.24 –.00)	.00 (-.12 –.12)	-.08 (-.20 –.04)	-.01 (-.13 –.11)	**-.32** (-.43 –-.20)
Intrusive	**.36** (.25 –.46)	.20 (.08 –.32)	**.31** (.19 –.42)	**.33** (.22 –.44)	.01 (-.11 –.13)
***Mean Alter Characteristics*** (k = 1, N = 148)					
Self-Centered	**.33**^a^ (.18 –.47)	.04^b^ (-.12 –.20)	.19 (.03 –.34)	.24 (.08 –.39)	**.34** (.19 –.48)
Attractive	-.05^a^ (-.21 –.11)	.21^b^ (.05 –.36)	-.03 (-.19 –.13)	.00 (-.16 –.16)	-.21 (-.36 –-.05)
Social Status	-.12 (-.28 –.04)	.09 (-.07 –.25)	-.05 (-.21 –.11)	-.10 (-.26 –.06)	-.16 (-.31 –.00)
Leadership	-.04^a^ (-.20 –.12)	.20^b^ (.04 –.35)	-.02 (-.18 –.14)	.00 (-.16 –.16)	-.13 (-.29 –.03)
Narcissism	.29^a^ (-.13 –.43)	.06^b^ (-.10 –.22)	.16 (.00 –.31)	.21 (.05 –.36)	**.31** (.16 –.45)
Intelligence	-.17^a^ (-.32 –-.01)	.21^b^ (.05 –.36)	-.11 (-.27 –.05)	-.14 (-.29 –.02)	-.17 (-.32 –-.01)
Likeable	-.22^a^ (-.37 –-.06)	.18^b^ (.02 –.33)	-.12 (-.28 –.04)	-.15 (-.30 –.01)	-.25 (-.40 –-.09)
Kindness	-.27^a^ (-.41 –-.11)	.18^b^ (.02 –.33)	-.18 (-.33 –-.02)	-.14 (-.29 –.02)	-.29 (-.43 –-.13)
***Mean Alter Personality*** (k = 1, N = 148)					
Neuroticism	.18^a^ (.02 –.33)	-.05^b^ (-.21 –.11)	.07 (-.09 –.23)	.13 (-.03 –.29)	.25 (.09 –.40)
Extraversion	-.09^a^ (-.25 –.07)	.18^b^ (.02 –.33)	.05 (-.11 –.21)	-.11 (-.27 –.05)	-.22 (-.37 –-.06)
Openness to Experience	-.05 (-.21 –.11)	.11 (-.05 –.27)	-.04 (-.20 –.12)	.01 (-.15 –.17)	-.14 (-.29 –.02)
Agreeableness	-.24^a^ (-.39 –-.08)	.20^b^ (.04 –.35)	-.10 (-.26 –.06)	-.09 (-.25 –.07)	**-.37** (-.50 –-.22)
Conscientiousness	-.16^a^ (-.31 –.00)	.18^b^ (.02 –.33)	-.11 (-.27 –.05)	-.13 (-.29 –.03)	-.20 (-.35 –-.04)
***Mean Relationship Dynamics*** (k = 1, N = 148)					
Frequency of Arguing	.24^a^ (.08 –.39)	-.14^b^ (-.29 –.02)	.18 (.02 –.33)	.12 (-.04 –.28)	.18 (-.02 –.33)
Envying the Alter	.05^a^ (-.1 –.21)	-.24^b^ (-.39 –-.08)	.09 (-.07 –.25)	.04 (-.12 –.20)	.08 (-.08 –.24)
Comparing Self to Alter	.13^a^ (-.03 –.29)	-.14^b^ (-.29 –.02)	.11 (-.05 –.27)	.12 (-.04 –.28)	.06 (-.10 –.22)
Closeness	-.01 (-.17 –.15)	.18 (.02 –.33)	.10 (-.06 –.26)	-.04 (-.20 –.12)	-.21 (-.36 –-.05)
Familiarity	-.09^a^ (-.25 –.07)	.22^b^ (.06 –.37)	.00 (-.16 –.16)	-.06 (-.22 –.10)	-.24 (-.39 –-.08)

*Note*. Bold = Effect sizes ≥.30; () = 95% Confidence Interval; LA = Leadership/Authority subscale of the NPI; GE = Grandiose/Exhibitionism subscale of the NPI; EE = Entitlement/Exploitativeness subscale of the NPI

^ab^superscripts are used to indicate that Steiger’s z-tests suggest these correlations are significantly different from one another.

Each NPI subscale demonstrated positive correlations with experiencing and expressing anger, as well directly confrontational responses such as yelling, threatening, and implementing physical aggression, with LA and EE bearing correlations of medium magnitude (Table 3). Additionally, each narcissism subscale was positively related to a drive for acquisition in a resource-sharing dilemma, as well as bids for disproportionate resources.

In terms of the interpersonal circumplex, self-esteem and total narcissism were both positively related to dominant and gregarious-extraverted interpersonal styles, and negatively related to unassured-submissive and unassuming-ingenuous styles. Narcissism bore uniquely positive relations to a cold-hearted interpersonal style, and a uniquely negative relation to a warm-agreeable style. Additionally, narcissism was associated with significantly more interpersonal problems than self-esteem, including appearing domineering, vindictive, and intrusive, though self-esteem was also positively correlated with these variables.

The NPI LA and GE subscales were both positively related to dominant and gregarious-extraverted interpersonal styles, and negatively related to unassured-submissive and unassuming-ingenuous styles. NPI EE was unrelated to dominance, but was negatively related to love and a warm-agreeable style. All of the narcissism subscales were associated with arrogant-calculating tendencies. Although LA and GE were positively associated with a gregarious-extraverted style, EE was negatively related. Additionally, all of the subscales were positively related to domineering and vindictive interpersonal problems, and negatively related to unassertive and exploitable approaches. NPI LA and GE subscales were uniquely associated with socially-avoidant (-) and intrusive (+) interpersonal problems, whereas the NPI EE subscale bore unique, negative relations to overly-nurturant interpersonal problems.

Regarding perceptions of one’s social networks, total narcissism was positively related to feeling central to one’s network, but self-esteem was unrelated to this perception. Self-esteem was positively related to perceiving individuals in one’s social network as attractive, high status, high in leadership, intelligent, likeable, and kind, whereas narcissism was negatively related to each of these perceptions. Narcissism was also significantly more strongly related to perceiving others in one’s network as narcissistic. Additionally, narcissism was related to perceiving peers as neurotic, disagreeable, and disinhibited, whereas the opposite was true for self-esteem. Narcissism was also related to more frequent arguing and social comparisons than was self-esteem, and self-esteem was uniquely positively related to feeling close to those in one’s network. Narcissism and self-esteem significantly differed on 38/51 (75%) of the interpersonal functioning variables assessed.

All of the NPI subscales were related to seeing others in one’s social network in negative light, including perceiving one’s network as self-centered and narcissistic, as well unintelligent, unlikeable, and unkind. NPI EE bore small-to-medium relations to of each of these perceptions and was also related to viewing the others in one’s social network as disagreeable, unconscientious, introverted, and neurotic. NPI EE was also related to not feeling close or familiar to those in one’s network, and all of the subscales were related to arguing frequently.

#### Psychopathology

Although total narcissism was weakly negatively related to anxiety and depression, self-esteem manifested strong, negative relations to these domains of psychopathology (Table 4). Similarly, the relation between self-esteem and global distress was strongly negative, whereas the narcissism-distress relation was negative but small. With regard to the PAI-based indices of psychopathology, total narcissism was positively related to most constructs, especially mania, antisocial behavior, and aggression, whereas self-esteem was unrelated or weakly negatively related to these constructs. Self-esteem showed a uniquely strong relation with treatment rejection, though it is possible that this is due to a lack of reported symptoms or distress (i.e., not needing treatment). Narcissism and self-esteem significantly differed on 17/17 (100%) of the internalizing psychopathology variables assessed.

**Table 4:** Internalizing and externalizing psychopathology.

	Narcissism	Self-Esteem	NPI-LA	NPI-GE	NPI-EE
***PAI*** (k = 1, N = 270)	*r*	*r*	*r*	*r*	*r*
Somatic Complaints	.20^a^ (.08 –.31)	-.28^b^ (-.39 –-.17)	.17 (.05 –.28)	.09 (-.03 –.21)	.23 (.11 –.34)
Anxiety	.11^a^ (-.01 –.23)	**-.41**^b^ (-.50 –-.31)	.02 (-.10 –.14)	-.01 (-.13 –.11)	.29 (.18 –.40)
Anxiety Disorders	.15^a^ (.03 –.26)	**-.35**^b^ (-.45 –-.24)	.10 (-.02 –.22)	.00 (-.12 –.12)	.27 (.16 –.38)
Depression	.00^a^ (-.12 –.12)	**-.48**^b^ (-.57 –-.38)	-.03 (-.15 –.09)	-.14 (-.26 –-.02)	.24 (.12 –.35)
Mania	**.56**^a^ (.47 –.64)	.01^b^ (-.11 –.13)	**.46** (.36 –.55)	**.41** (.31 –.50)	**.39** (.28 –.35)
Paranoia	.24^a^(.12 –.35)	-.26^b^ (-.37 –-.15)	.21 (.09 –.32)	.05 (-.07 –.17)	**.39** (.28 –.49)
Schizophrenia	.14^a^ (.02 –.26)	**-.32**^b^ (-.42 –-.21)	.11 (-.01 –.23)	.01 (-.11 –.13)	**.32** (.21 –.42)
Borderline	.18^a^ (.06 –.29)	**-.42**^b^ (-.51 –-.32)	.09 (-.03 –.21)	.10 (-.02 –.22)	**.31** (.20 –.41)
Antisocial	**.38**^a^ (.27 –.48)	-.11^b^ (-.23 –.01)	.26 (.15 –.37)	.29 (.18 –.40)	**.34** (.23 –.44)
Alcohol	.25^a^ (.13 –.36)	-.16^b^ (-.27 –.04)	.14 (.02 –.26)	.22 (.10 –.33)	.29 (.18 –.40)
Drugs	.19^a^ (.07 –.30)	-.14^b^ (-.26 –-.02)	.12 (.00 –.24)	.16 (.04 –.27)	.24 (.12 –.35)
Aggression	**.44**^a^ (.34 –.53)	-.11^b^ (-.23 –.01)	**.35** (.24 –.45)	**.30** (.19 –.40)	**.45** (.35 –.54)
Suicide	.06^a^ (-.06 –.18)	**-.40**^b^ (-.50 –-.29)	.02 (-.10 –.14)	.03 (-.09 –.15)	.18 (.06 –.29)
Treatment Rejection	.10^a^ (-.02 –.22)	**.39**^b^ (.28 –.49)	.15 (.03 –.26)	.07 (-.05 –.19)	-.06 (-.18 –.06)
***BSI*** (k = 4, N = 1018)					
Global Distress	-.10^a^ (-.16 –-.03)	**-.52**^b^ (-.56 –-.47)	-.16 (-.22 –-.10)	-.07 (-.13 –-.01)	.17 (.11 –.24)
***Promis*** (k = 3, N = 1841)					
Anxiety	-.08^a^ (-.16 –.01)	**-.64**^a^ (-.66 –-.60)	-.17 (-.24 –-.10)	-.10 (-.16 –-.03)	.09 (-.05 –.23)
Depression	-.14^a^ (-.22 –-.06)	**-.68**^a^ (-.81 –-.50)	-.23 (-.29 –-.17)	-.13 (-.20 –-.07)	.07 (-.09 –.21)
***Affect*** (k = 3, N = 1655)					
Negative Affect	.02^a^ (-.05 –.08)	**-.49**^b^ (-.53 –-.37)	-.03 (-.11 –.05)	.05 (.00 –.11)	.19 (.11 –.28)
Positive Affect	**.30** (.25 –.35)	**.46** (.36 –.55)	**.32** (.25 –.38)	.21 (.16 –.25)	-.04 (-.08 –.01)
***Externalizing***					
Alcohol Use^1^	.16^a^ (-.11 –.21)	-.06^b^ (-.10 –-.01)	.08 (.03 –.14)	.15 (.10 –.20)	.11 (.06 –.16)
Substance Use^2^	.10^a^ (.03 –.16)	-.04^b^ (-.08 –-.01)	.02 (-.02 –.07)	.02 (-.06 –.09)	.01 (-.04 –.05)
Antisocial Behavior^2^	.19^a^ (.13 –.25)	-.05^b^ (-.08 –-.02)	.12 (.08 –.17)	.07 (.03 –.12)	.13 (.09 –.18)
***Self-Report Aggression*** (k = 2, N = 815)					
Reactive	.20^a^ (.11 –.28)	-.17^b^ (-.31 –-.01)	.15 (.07 –.24)	.11 (.05 –.18)	.26 (.19 –.32)
Proactive	.27^a^ (.20 –.33)	-.08^b^ (-.17 –-.01)	.17 (.10 –.24)	.21 (.10 –.32)	**.35** (.23 –.46)
Total	.24^a^ (.17 –.30)	-.10^b^ (-.17 –-.03)	.17 (.10 –.24)	.19 (.12 –.26)	.29 (.22 –.35)
***Lab Aggression*** (k = 1, N = 220)					
Total Aggression	.18 (.04 –.30)	.06 (-.07 –.19)	.11 (-.02 –.24)	.11 (-.02 –.24)	.17 (.04 –.29)

*Note*. Bold = Effect sizes ≥.30; () = 95% Confidence Interval

^1^ = k = 3, N = 1609

^2^ = k = 3, N = 3197; LA = Leadership/Authority subscale of the NPI; GE = Grandiose/Exhibitionism subscale of the NPI; EE = Entitlement/Exploitativeness subscale of the NPI

^ab^superscripts are used to indicate that Steiger’s z-tests suggest these correlations are significantly different from one another.

Narcissism and self-esteem also showed divergent relations with externalizing behavior. Although narcissism was positively related to alcohol/substance use, antisocial behavior, and aggression (self-reported and laboratory-based), self-esteem evinced null or negative correlations with these variables. Narcissism and self-esteem significantly differed on 6/7 (86%) of the externalizing psychopathology variables assessed.

With regard to the PAI-based indices of psychopathology, NPI EE was positively related to nearly every construct, especially aggression, mania, and paranoia (Table 4) whereas NPI LA and GE demonstrated more specific relation to mania, aggression, and antisocial behavior. With regard to affective experiences, NPI LA and GE bore unique, positive relations to positive affect, whereas NPI EE showed unique positive relations to negative affect and global distress. In terms of externalizing behavior, each narcissism subscale was associated with self-report and lab aggression, and EE bore the strongest correlates, although these relations were small-to-medium in nature. Each subscale was also positively correlated with alcohol use, substance use, and antisocial behavior, though these relations were small in nature.

#### Pathological traits

Both total narcissism and self-esteem were positively correlated with entitlement, but this relation was large for narcissism and small for self-esteem (Table 5). Large differences were present in relations with the DSM-5 pathological traits as measured by the PID-5. Total narcissism was related to a higher score on *every* pathological trait compared to self-esteem. In fact, narcissism showed positive correlations with 27/30 pathological traits, whereas self- esteem manifested negative correlations with 30/30 traits. Self-esteem was strongly negatively associated with negative affectivity, detachment, disinhibition, and psychoticism, but narcissism showed either small positive (negative affectivity, detachment) or medium positive (disinhibition, psychoticism) relations with these traits. Finally, narcissism evinced a strong, positive relation with antagonism, but self-esteem’s relation to antagonism was moderate and negative. Narcissism and self-esteem significantly differed on 26/26 (100%) of the pathological trait variables assessed.

**Table 5:** Pathological traits.

	Narcissism	Self-Esteem	NPI-LA	NPI-GE	NPI-EE
***PID-5*** (k = 1, N = 306)	*r*	*r*	*r*	*r*	*r*
**Negative Affectivity**	.15^a^ (.04 –.26)	**-.68**^**b**^ (-.74 –-.61)	.00 (-.11 –.11)	.18 (.07 –.29)	**.38** (.28 –.47)
Emotional Lability	.17^a^ (.06 –.28)	**-.51**^**b**^ (-.59 –.42)	.06 (-.05 –.17)	.19 (.08 –.30)	.28 (.17 –.38)
Anxiousness	-.10^a^ (-.21 –.01)	**-.66**^**b**^ (-.72 –-.59)	-.17 (-.28 –-.06)	-.05 (-.16 –.06)	.21 (.10 –.31)
Separation Insecurity	.25^a^ (.14 –.35)	**-.41**^**b**^ (-.50 –-.31)	.07 (-.04 –.18)	**.33** (.23 –.43)	.29 (.18 –.39)
Submissiveness	.03^a^ (-.08 –.14)	**-.38**^**b**^ (-.47 –-.28)	.01 (-.10 –.12)	.07 (-.04 –.18)	.11 (.00 –.22)
Hostility	.29^a^ (.18 –.39)	**-.42**^**b**^ (-.51 –-.32)	.17 (.06 –.28)	.26 (.15 –.36)	**.46** (.37 –.54)
Perseveration	.10^a^ (-.01 –.21)	**-.56**^**b**^ (-.63 –-.48)	.00 (-.11 –.11)	.13 (.02 –.24)	**.32** (.22 –.42)
**Detachment**	.06^a^ (-.05 –.17)	**-.64**^**b**^ (-.70 –-.57)	-.07 (-.18 –.04)	.08 (-.03 –.19)	**.35** (.25 –.44)
Withdrawal	-.01^a^ (-.12 –.10)	**-.33**^**b**^ (-.43 –-.23)	-.05 (-.16 –.06)	-.09 (-.20 –.02)	.24 (.13 –.34)
Intimacy Avoidance	.11^a^(.00 –.22)	**-.36**^**b**^ (-.45 –-.26)	-.02 (-.13 –.09)	.10 (-.01 –.21)	**.30** (.19 –.40)
Anhedonia	-.08^a^ (-.19 –.03)	**-.71**^**b**^ (-.76 –-.65)	-.20 (-.31 –-.09)	-.02 (-.13 –.09)	.28 (.17 –.38)
Depressivity	.04^a^ (-.07 –.15)	**-.76**^**b**^ (-.80 –-.71)	-.11 (-.22 –.00)	.10 (-.01 –.23)	**.32** (.22 –.42)
Suspiciousness	.20^a^ (.09 –.31)	**-.45** (-.54 –-.36)	.06 (-.05 –.17)	.20 (.09 –.31)	**.33** (.23 –.43)
(Lack of) Restricted Affectivity	.15^a^ (.04 –.26)	-.19^**b**^ (-.30 –-.08)	.07 (-.04 –.18)	.12 (.01 –.23)	.19 (.08 –.30)
**Antagonism**	**.50**^a^ (.41 –.58)	**-.36**^**b**^ (-.45 –-.26)	**.31** (.20 –.41)	**.44** (.34 –.53)	**.54** (.46 –.61)
Manipulativeness	**.49**^a^ (.40 –.57)	-.18^**b**^ (-.29 –-.07)	**.34** (.24 –.44)	**.38** (.28 –.47)	**.42** (.32 –.51)
Deceitfulness	**.39**^a^ (.29 –.48)	**-.37**^**b**^ (-.46 –-.27)	.22 (.11 –.32)	**.32** (.22 –.42)	**.46** (.37 –.54)
Grandiosity	**.57**^a^ (.49 –.64)	-.08^**b**^ (-.19 –.03)	**.39** (.29 –.48)	**.48** (.39 –.56)	**.41** (.31 –.50)
Attention Seeking	**.57**^a^ (.49 –.64)	-.17^**b**^ (-.28 –-.06)	**.40** (.30 –.49)	**.57** (.49 –.64)	**.39** (.29 –.48)
Callousness	**.35**^a^ (.25 –.44)	**-.41**^**b**^ (-.50 –-.31)	.19 (.08 –.30)	**.30** (.19 –.40)	**.50** (.41 –.58)
**Disinhibition**	**.32**^a^ (.22 –.42)	**-.47**^**b**^ (-.55 –-.38)	.15 (.04 –.26)	**.32** (.22 –.42)	**.44** (.34 –.53)
Irresponsibility	.28^a^ (.17 –.38)	**-.48**^**b**^ (-.56 –-.39)	.06 (-.05 –.17)	**.31** (.20 –.41)	**.48** (.39 –.56)
Impulsivity	.26^a^ (.15 –.36)	**-.40**^**b**^ (-.49 –-.30)	.09 (-.02 –.20)	**.32** (.22 –.42)	**.39** (.29 –.48)
Distractibility	.06^a^ (-.05 –.17)	**-.59**^**b**^ (-.66 –-.51)	-.05 (-.16 –.06)	.10 (-.01 –.21)	**.36** (.26 –.48)
Risk Taking	**.35**^a^ (.25 –.44)	-.05^**b**^ (-.16 –.06)	.26 (.15 –.36)	.28 (.17 –.38)	.15 (.04 –.26)
(Lack of) Rigid Perfectionism	.20^a^ (.09 –.31)	-.23^**b**^ (-.33 –-.12)	.14 (.03 –.25)	.16 (.05 –.27)	.26 (.15 –.36)
**Psychoticism**	.26^a^ (.15 –.36)	**-.46**^**b**^ (-54 –-.37)	.13 (.02 –.24)	.21 (.10 –.31)	**.40** (.30 –.49)
Unusual Beliefs	**.37**^a^ (.27 –.46)	-.27^**b**^ (-.37 –-.16)	.25 (.14 –.35)	.29 (.18 –.39)	**.38** (.28 –.47)
Eccentricity	.08^a^ (-.03 –.19)	**-.45**^**b**^ (-.54 –-.36)	.01 (-.10 –.12)	.04 (-.07 –.15)	**.30** (.19 –.40)
***Entitlement*** (k = 7, N = 2867)	**.49**^a^ (.39 –.58)	.09^**b**^ (.05 –.13)	.14 (.03 –.25)	**.30** (.19 –.40)	**.41** (.31 –.50)

*Note*. Bold = Effect sizes ≥ .30; () = 95% Confidence Interval; LA = Leadership/Authority subscale of the NPI; GE = Grandiose/Exhibitionism subscale of the NPI; EE = Entitlement/Exploitativeness subscale of the NPI

^ab^superscripts are used to indicate that Steiger’s z-tests suggest these correlations are significantly different from one another.

With regard to the narcissism subscales, NPI EE subscale showed consistent medium to-large correlations with every pathological trait domain as measured by the PID-5 (Table 5). All three NPI subscales demonstrated medium to strong relations to antagonism, but NPI EE evinced uniquely strong relations to negative affectivity, detachment, and psychoticism. The GE and EE subscales also showed medium-to-large relations to disinhibition, whereas LA’s relation to this domain was relatively small. Additionally, all three narcissism subscales were related to entitlement, although the relations were largest for NPI EE.

#### Personality disorders

Total narcissism manifested positive correlations with Cluster B personality disorders, most strongly with narcissistic and histrionic personality disorders (Table 6). Self-esteem was either unrelated or negatively related (effect sizes ranging from small to large) to every personality disorder. Narcissism was positively related to 8/10 personality disorders. Additionally, narcissism showed expected, positive relations to Machiavellianism, grandiose narcissism and psychopathy. Self-esteem showed a small-moderate correlation with grandiose narcissism, but this relation was much weaker than that between narcissism and grandiose narcissism. Finally, narcissism showed a small, positive relation with vulnerable narcissism, whereas self-esteem showed a moderate, negative relation. Narcissism and self-esteem significantly differed on 14/14 (100%) of the personality disorder variables assessed.

**Table 6:** Personality disorders.

	Narcissism	Self-Esteem	NPI-LA	NPI-GE	NPI-EE
***DSM-IV PDs*** (k = 3, N = 1502) *r*		*r*	*r*	*r*	*r*
Paranoid	.17^a^ (.12 –.22)	-.28^b^ (-.33 –-.23)	.08 (-.01 –.17)	.07 (-.01 –.16)	**.37** (.29 –.44)
Schizoid	.05^a^ (-.05 –.16)	-.19^b^ (-.25 –.14)	-.01 (-.10 –.08)	-.07 (-.15 –.02)	.15 (.03 –.25)
Schizotypal	.09^a^ (-.02 –.20)	-.26^b^ (-.30 –-.22)	-.01 (-.12 –.10)	-.02 (-.11 –.06)	.12 (-.04 –.29)
Antisocial	.28^a^ (.24 –.33)	-.14^b^ (-.19 –-.09)	.18 (-.09 –.26)	.25 (.17 –.33)	.25 (.17 –.33)
Borderline	.09^a^ (.01 –.16)	**-.43**^b^ (-.46 –-.39)	-.04 (-.13 –.05)	-.07 (-.16 –.02)	.26 (.17 –.34)
Histrionic	**.41**^a^ (.35 –.47)	.01^b^ (-.04 –.06)	.25 (.16 –.33)	**.53** (.47 –.59)	.25 (.17 –.34)
Narcissistic	**.40**^a^ (.36 –.44)	-.11^b^ (-.18 –-.04)	.27 (.20 –.35)	**.31** (.23 –.39)	**.46** (.30 –.59)
Avoidant	-.30^a^ (-.40 –-.20)	**-.47**^b^ (-.52 –-.41)	**-.38** (-.45 –-.30)	**-.31** (-.41 –-.21)	.06 (-.06 –.18)
Dependent	-.12^a^ (-.24 –.01)	**-.34**^b^ (-.38 –-.29)	-.27 (-.35 –-.18)	-.09 (-.18 –.00)	.05 (-.05 –.15)
Obsessive-Compulsive	.04^a^ (-.03 –.10)	-.13^b^ (-.18 –-.07)	-.01 (-.10 –.07)	-.09 (-.18 –.00)	.04 (-.05 –.13)
***Dark Triad***					
Psychopathy^1^	**.42**^a^ (.34 –.49)	-.14^b^ (-.23 –-.04)	**.32** (.20 –.44)	.25 (.17 –.34)	**.48** (.45 –.51)
Grandiose Narcissism^2^	**.71**^a^ (.54 –.82)	.23^b^ (.15 –.32)	**.62** (.48 –.73)	**.53** (.37 –.66)	**.48** (.27 –.64)
Vulnerable Narcissism^3^	.10^a^ (.04 –.17)	**-.37**^b^ (-.43 –-.32)	.00 (-.05 –.05)	.12 (.05 –.19)	**.33** (.26 –.38)
Machiavellianism^4^	**.31**^a^ (.22 –.40)	-.20^b^ (-.26 –-.15)	.29 (.23 –.34)	.24 (.18 –.30)	**.45** (.40 –.49)

*Note*. Bold = Effect sizes ≥ .30; () = 95% Confidence Interval

^1^k = 5, N = 2919

^2^k = 5, N = 1572

^3^k = 9, N = 4220

^4^k = 2, N = 2052; LA = Leadership/Authority subscale of the NPI; GE = Grandiose/Exhibitionism subscale of the NPI; EE = Entitlement/Exploitativeness subscale of the NPI

^ab^superscripts are used to indicate that Steiger’s z-tests suggest these correlations are significantly different from one another.

With regard to the narcissism subscales, NPI EE subscale showed positive relations to every DSM-IV personality disorder (PD), but these relations varied in magnitude (Table 6) with the largest relations for narcissistic, paranoid, and borderline PDs. NPI LA and GE also showed medium-to-large positive relations to narcissistic and histrionic PDs, and negatively related to Cluster C PDs, especially avoidant PD. Each of the subscales showed positive relations to the psychopathy, grandiose narcissism, and Machiavellianism. EE was the strongest correlate of psychopathy and Machiavellianism, and it was also correlated positively with vulnerable narcissism while the others showed weak or null relations.

#### Profile similarity

In order to examine the overall profile similarity between total narcissism and self-esteem, we calculated intraclass correlations for all of the variables in Tables 1–6 [112]. Overall, the profiles associated with total narcissism and self-esteem were unrelated (*r*_*ICC*_ = -.05). At the subscale level, only the NPI LA factor manifested a positive, albeit small, degree of similarity to self-esteem (*r*_ICC_ = .18). The NPI GE subscale was unrelated to self-esteem (*r*_ICC_ = -.05), whereas NPI EE subscale and self-esteem were strongly negatively related (*r*_ICC_ = -.59).

To examine which criteria proffered the most similar and divergent correlations with narcissism and self-esteem, we computed and rank-ordered the absolute value of the difference between narcissism and self-esteem’s correlation with each variable (Table 7). The largest differences were in relation to DSM-5 personality disorder trait domains, where narcissism and self-esteem were remarkably different. Other large differences were present in narcissism and self-esteem’s relations to antagonism-related variables such as psychopathy, aggression, and (low) agreeableness. Alternately, narcissism and self-esteem showed similar relations to extraversion (+), behavioral inhibition (-), and positive affect (+).

**Table 7:** 15 Largest and smallest differences between narcissism and self-esteem.

Largest		Smallest^1^	
Variable	Difference (Narc, SE)^2^	Variable	Difference (Narc, SE)^2^
PID Antagonism^3^	.86 (.50, -.36)	Extraversion (self)	.03 (.42, .39)
PID Negative Affectivity	.83(.15, -.68)	Behavior Inhibition	.03 (-.30, -.33)
PID Disinhibition	.79 (.32, -.47)	Likeability (thin slice)	.04 (.20, .24)
PID Psychoticism	.72 (.26, -.46)	Neuroticism (thin slice)	.04 (-.29, -.25)
PID Detachment	.70 (.06, -.64)	IAS Unassuming-Ingenuous	.05 (-.34, -.29)
PAI Borderline	.60 (.18, -.42)	IAS Dominance	.05 (.53, .58)
IAS Cold-Hearted	.59 (.42, -.17)	Extraversion (informant)	.07 (.23, .16)
Psychopathy	.56 (.42, -.14)	IAS Assured-Dominant	.10 (.53, .43)
Promis Anxiety	.56 (-.08, -.64)	Extraversion (thin slice)	.10 (.40, .30)
PAI Mania	.55 (.56, .01)	IIP Socially Avoidant	.11 (-.39, -.28)
PAI Aggression	.55 (.44, -.11)	IAS Unassured-Submissive	.11 (-.38, -.49)
Promis Depression	.54 (-.14, -.68)	BIS/BAS Fun-Seeking	.11 (.22, .11)
Agreeableness (self)	.53 (-.42, .11)	Attractiveness (thin slice)	.11 (.23, .12)
Borderline PD	.52 (.09, -.43)	BIS/BAS Reward Responsiveness	.15 (.09, .24)

Note.

^1^to be considered for this compilation, one of the correlations between narcissism or self-esteem and the other variable must be at least r = |.20|

^2^absolute value of the difference in Pearson’s *r* between narcissism and self-esteem (correlation with narcissism, self-esteem)

^3^for the sake of parsimony, we are only reporting the differences for the PID-5 domains, but note there are many large facet level differences

With regard to the largest and smallest differences between self-esteem and the NPI subscales (see Tables 8–10), each of the subscales demonstrated very different relations to each of the DSM-5 personality disorder trait domains. The LA subscale also proffered notably different relations to Borderline PD and Machiavellianism, but showed similar relations to Behavior Inhibition, Dominance, and Extraversion. The GE subscale demonstrated the most different relations to Depression and Neuroticism, and the most similar relations to Extraversion, Reward Responsiveness, and thin slice Likeability and Neuroticism. Finally, the EE subscale was most different than self-esteem in their relations to Neuroticism, Depression and Anxiety, and Borderline PD. Alternately, the EE subscale was most similar to self-esteem in their relations to Domineering interpersonal problems, Drive, and thin slice-assessed Extraversion and Neuroticism.

**Table 8:** 10 largest and smallest differences between self-esteem and the NPI LA subscale.

Largest		Smallest^1^	
Variable	Difference (SE, LA)^2^	Variable	Difference (SE, LA)^2^
PID Negative Affectivity	.68 (-.68, .00)	Behavior Inhibition	.01 (-.33, -.34)
PID Antagonism	.67 (-.36, .31)	IAS Dominance	.02 (.58, .56)
PID Disinhibition	.62 (-.47, .15)	IAS Unassuming-Ingenuous	.04 (-.29, -.33)
PID Psychoticism	.59 (-.46, .13)	Extraversion (self)	.05 (.39, .44)
PID Detachment	.57 (-.64, -.07)	Neuroticism (thin slice)	.05 (-.25, -.30)
Borderline PD	.51 (-.42, .09)	Entitlement	.05 (.09, .14)
Machiavellianism	.49 (-.20, .29)	IIP Socially-Avoidant	.07 (-.28, -.35)
Promis Anxiety	.47 (-.64, -.17)	IAS Unassured-Submissive	.07 (-.49, -.42)
PAI Paranoia	.47 (-.26, .21)	Dependent PD	.07 (-.34, -.27)
IAS Cold-Hearted	.47 (-.17, .30)	Alter Closeness	.08 (.18, .10)

Note.

^1^to be considered for this compilation, one of the correlations between narcissism or self-esteem and the other variable must be at least r = |.20|

^2^absolute value of the difference in Pearson’s *r* between narcissism and self-esteem (correlation with narcissism, self-esteem); for the sake of parsimony, we are only reporting the differences for the PID-5 domains, but note there are many large facet level differences.

**Table 9:** 10 largest and smallest differences between self-esteem and the NPI GE subscale.

Largest		Smallest^1^	
Variable	Difference (SE, GE)^2^	Variable	Difference (SE, GE)^2^
PID Negative Affectivity	.86 (-.68, .18)	Likeability (thin slice)	.00 (.24, .24)
PID Antagonism	.80 (-.36, .44)	Extraversion (self)	.02 (.39, .41)
PID Disinhibition	.79 (-.47, .32)	Reward Responsiveness	.03 (.24, .21)
PID Detachment	.72 (-.64, .08)	Neuroticism (thin slice)	.04 (-.25, -.29)
PID Psychoticism	.67 (-.46, .21)	IIP Unassertive	.05 (-.19, -.24)
Promis Depression	.55 (-.68, -.13)	IIP Domineering	.05 (.22, .27)
Neuroticism (self)	.55 (-.65, -.10)	IIP Socially Avoidant	.06 (-.28, -.34)
PANAS Negative Affect	.54 (-.49, .05)	IAS Unassuming-Ingenuous	.07 (-.29, -.22)
Promis Anxiety	.54 (-.64, -.10)	Extraversion (thin slice)	.12 (.30, .42)
Borderline PD	.52 (-.42, .10)	IAS Assured Dominant	.12 (.43, .31)

Note.

^1^to be considered for this compilation, one of the correlations between narcissism or self-esteem and the other variable must be at least r = |.20|

^2^absolute value of the difference in Pearson’s *r* between narcissism and self-esteem (correlation with narcissism, self-esteem); for the sake of parsimony, we are only reporting the differences for the PID-5 domains, but note there are many large facet level differences.

**Table 10:** 10 largest and smallest differences between self-esteem and the NPI EE subscale.

Largest		Smallest^1^	
Variable	Difference (SE, EE)^2^	Variable	Difference (SE, EE)^2^
PID Negative Affectivity	1.06 (-.68, .38)	IIP Domineering	.04 (.22, .26)
PID Detachment	.99 (-.63, .35)	Drive	.05 (.22, .17)
PID Disinhibition	.91 (-.47, .44)	Extraversion (thin slice)	.18 (.30, .12)
PID Antagonism	.90 (-.36, .54)	IAS Unassuming-Ingenuous	.18 (-.29, -.11)
PID Psychoticism	.86 (-.46, .40)	IIP Intrusive	.19 (.20, .01)
IAS Aloof-Introverted	.80 (-.51, .29)	Neuroticism (thin slice)	.20 (-.25, -.05)
Neuroticism (self)	.77 (-.65, .12)	IAS Arrogant-Calculating	.20 (.12, .32)
Promis Depression	.75 (-.68, .07)	IIP Vindictive	.20 (.11, .31)
Promis Anxiety	.73 (-.64, .09)	IIP Socially Avoidant	.20 (-.28, -.08)
Borderline PD	.73 (-.42, .31)	Likeability (thin slice)	.22 (.24, .02)

Note.

^1^to be considered for this compilation, one of the correlations between narcissism or self-esteem and the other variable must be at least r = |.20|

^2^absolute value of the difference in Pearson’s *r* between narcissism and self-esteem (correlation with narcissism, self-esteem); for the sake of parsimony, we are only reporting the differences for the PID-5 domains, but note there are many large facet level differences.

## Discussion

In the psychology literature and in everyday discourse, narcissism and self-esteem are two primary terms to describe relatively stable (i.e., trait) self-positivity. Despite this conceptual overlap, the constructs are far from identical, as self-esteem is robustly associated with mental health, whereas narcissism can be diagnosed as a personality disorder at its extremes. By examining the empirical profiles associated with both constructs, we can understand precisely where grandiose narcissism, and the components that comprise it, and explicit self-esteem converge and diverge. Across a combined sample of 4711 (k = 11), we found a small to moderate, positive correlation between the constructs (*r =* .28), as measured by the two most commonly used assessments of both. A quantitative analysis of the similarity of their nomological networks based on the entire assortment of criteria we examined–including several basic models of personality, retrospective reports of developmental experiences, interpersonal functioning, and psychopathology, there was essentially no correlation between the two. Although similar analyses suggested that the nomological network of the NPI LA subscale, which is generally associated with agentic traits, shows a small, positive relation to self-esteem’s nomological network, the nomological network of the NPI EE subscale showed a large, negative relation to that of self-esteem. Taken together, these analyses suggest that the subcomponents of narcissism show relations to self-esteem that vary quite substantially in magnitude and direction: at their most similar, they demonstrate a small, positive relation, but at their most distinct, they demonstrate a large, negative relation.

### Agency and communion

We hypothesized two main differences between narcissism and self-esteem: 1) their relations to variables related to agreeableness/communion, and 2) their relations to pathological intra- and interpersonal functioning. In terms of the first hypothesis, self-esteem and narcissism share a fundamental core of an approach-oriented, agentic and extraverted interpersonal style. In fact, the relations between self-esteem and narcissism are essentially the same between self-report, informant-report, and thin slice extraversion (+), behavioral inhibition (-), and a dominant interpersonal style (+). Importantly, this similarity is most evident when examining the relations of the NPI LA and GE subscales but not the EE subscale, which is consistent with prior reported relations. The homogeneity of results across samples, methodologies, and subscales underscores the centrality of agency in accounting for where these constructs overlap.

Alternately, they diverge strongly in the communal domain, such that narcissism is uniquely characterized by an antagonistic, non-communal interpersonal style. For example, the largest difference between narcissism and self-esteem is in their relation to antagonism (Table 7), and other pronounced differences support this distinction (e.g., relations to psychopathy, self-report agreeableness, and a cold-hearted interpersonal style). As hypothesized, the NPI EE subscale was the subscale related most strongly to the communal domain, supporting the models of narcissism that include both a (high) agency and (low) communion component. This consistent pattern of findings is critical to understanding how narcissism and self-esteem function more broadly, as differences in communion/agreeableness are relevant to many important outcomes including relational health [115, 116] occupational functioning (e.g., organizational citizenship; [117]), and externalizing behavior such as antisocial behavior and aggression [50, 118].

A related way of conceptualizing this distinction is in terms of a “zero sum” social orientation, which is related to the adequacy/superiority distinction. As exemplified by narcissism’s positive relations to disproportionate bids for resources in a game context, this trait profile appears to be associated with a willingness to take more than one’s fair share in a situation where resources are limited. In other words, narcissism is related to an inclination to obtain an unfairly sizable (i.e., superior) share of resources than others, instead of an equal (i.e., adequate) one. Although we live in a world where certain “resources” are theoretically unlimited and available to all (e.g., friendship), the current data suggest that narcissistic individuals may be more inclined to exploit others even when resources are not necessarily limited. Furthermore, this exploitation is likely related to the tendencies of narcissistic individuals to view others in their social network as self-centered, unkind, and disagreeable, as these traits may be seen as commonplace and necessary to retain a sense of superiority and higher (perceived) social status.

### Functional outcomes

In terms of our second hypothesis, our data provide robust support for the role of self-esteem as an almost wholly adaptive construct. Self-esteem’s nomological network includes positive associations with a range of criteria that are generally considered adaptive and negative associations with a range of criteria that are generally considered maladaptive. For example, self-esteem was related to likability, as well as the tendency to describe others in one’s social network as attractive, smart, and kind. Importantly, self-esteem also bore strong, negative relations to adverse developmental experiences, pathological traits, and psychopathology. A key component of self-esteem is its strong relation to emotional stability, which appears to act as a protective factor against a range of maladaptive outcomes. Overall, self-esteem is characterized by robust psychological adjustment across an array of domains.

Of course, the current data do not speak to whether self-esteem is a mere correlate of psychological health or if it is causally related; if it is causally related, the direction of this relation cannot be tested from these data (e.g., psychological health leads to good self-esteem or good self-esteem leads to good psychological health). In a comprehensive review, Baumeister and colleagues [119] reviewed multiple longitudinal studies investigating the relationship between success in a number of domains and self-esteem. Although the authors reported mixed results [120, 121], there was modest evidence that self-esteem was an outcome, rather than a cause, of academic performance. These results were generally very small, however, and the most important conclusion from this review is that a causal relation between self-esteem and success (in either direction) has generally not been established. Some authors have concluded that a third variable such as ability (e.g., IQ) may be an underlying causal factor [122]. The argument that self-esteem plays a causal role in adaptive outcomes is stronger for depression, where researchers have made a case that low self-esteem is a significant risk factor for depression [123].

Alternately, narcissism’s nomological network is more complex and characterized by signs of both adaptivity and maladaptivity. Narcissism is related to a range of more antagonism-oriented constructs including general and pathological trait antagonism, a domineering and vindictive interpersonal style, denigration of those closest to them in their social networks, and a propensity towards aggression, particularly the NPI EE subscale. Importantly, narcissism did not show the large negative associations with psychopathology and pathological traits exhibited by self-esteem, even when examining the NPI LA and GE subscales, which are more associated with agentic traits. Although narcissism is either unrelated or slightly negatively related to some forms of internalizing psychopathology, it is strongly related to an antagonistic interpersonal style characterized by deceitfulness, manipulativeness, attention seeking, grandiosity, entitlement, and callousness. Indeed, self and informant reports demonstrate that narcissistic individuals behave in a disagreeable manner (and this trait is even picked up by strangers via thin slice ratings). Furthermore, narcissism bore almost exclusively positive relations to pathological traits, whereas self-esteem bore exclusively negative relations.

Based on this nomological network, narcissism represents a construct that has both adaptive and maladaptive associations. Our results not only support the notion that agentic traits like extraversion are a large component of narcissism, at least when conceptualized as grandiose narcissism and assessed with the NPI, but that this trait can be detected very quickly and is associated with likeability. This quality is instrumental in helping narcissistic individuals achieve signs of status (e.g., leadership positions; [124]), and is also associated with subjective well-being [125]. Coupled with small, negative relations to internalizing psychopathology, especially the NPI subscales that capture a more agentic approach, narcissism can appear to be largely associated with psychological adjustment, at least when discussed in the context of internalizing, distress-based disorders. However, narcissism’s strong relation to low communion, especially the NPI EE subscale, which is most strongly associated with antagonism and a wide range of constructs that are generally considered socially undesirable (e.g., aggression), suggests important areas of dysfunction, particularly that of an interpersonal and externalizing form.

### Conclusions, limitations, and future directions

We conclude by revisiting the three aforementioned theoretical streams that are important in situating the current data in the wider context of the literature on narcissism. First, although we did not specifically test domain-specific self-positivity, our findings support the implications of the agency model that positive relations with agentic, but not communal, traits, are pertinent to narcissism. Both narcissism and self-esteem were strongly related to agentic traits (e.g., extraversion, dominance), but narcissism bore unique, strong, negative relations to communal traits (e.g., agreeableness, warmth). This is particularly underscored by the large relations between EE, the NPI subscale most strongly associated with antagonism, and multiple indicators of low communality, including traits, social responding, interpersonal problems, and externalizing psychopathology. Although self-esteem bore small relations with communal traits, they were consistently positive. Of course, our data are not well-suited to address the dynamics of how agentic traits may function differently for narcissistic individuals versus individuals with high self-esteem in reaction to inter- or intrapersonal events. We encourage future research into this area in which a more dynamic, granular approach is used (e.g., ecological momentary assessments).

Second, our data support the previous findings about the psychological health correlates of self-esteem. Previous work has shown strong, positive relations between self-esteem and happiness [119], and the current data complement this finding by demonstrating that self-esteem is strongly, negatively related to internalizing psychopathology. On the other hand, while total narcissism and the NPI subscales generally demonstrated small, null-to-negative relations to internalizing psychopathology, they were significantly smaller in magnitude, and narcissism was also related to a range of externalizing psychopathology variables. Thus, although we can make no causal claims, our data support that self-esteem is strongly related to psychological health (or at the very least, a lack of psychopathology), whereas narcissism appears to lose any links to psychological health once its shared variance with self-esteem is removed [21].

Finally, our data support a trait-based model of narcissism, which proposes that narcissism is best understood as an amalgam of FFM domains, including agency (i.e., extraversion, admiration, grandiosity), and (low) communion (i.e., antagonism, rivalry, and entitlement). The current results are aligned with this model (Table 11), which can also be used to understand self-esteem as trait profile. Although narcissism and self-esteem share a common core of extraversion, narcissism is uniquely related to antagonism (i.e., low agreeableness). Lastly, both are related to emotional stability (i.e., low neuroticism), but this relation is much stronger for self-esteem. Although the current analyses are not able to speak directly to the role that these particular traits play in determining narcissism’s nomological net, the NPI subscale relations provide some insight. As aforementioned, EE is the subscale most associated with antagonism, and indeed it is generally the strongest correlate of antagonistic traits and externalizing behaviors. Similarly, the LA subscale (which is more associated with extraversion than EE) is more strongly related to traits such as drive, dominance, and positive affect, and profile comparison suggests that this subscale has a small, positive associated with self-esteem. Thus, we believe that the current results provide a basis for how subcomponents from other models of narcissism may relate to the criteria examined herein. Such a nuanced approach can be helpful in identifying the specific elements of narcissism associated with various outcomes of interest [126, 127].

**Table 11:** A trait-based comparison of narcissism and self-esteem.

Core Trait	Sample Constructs	Self-Esteem	Narcissism
Assertive Extraversion/ Agency	extraversion, dominance, positive affect	+	+
Agreeableness/ Communion	agreeableness, antagonism, aggression	+	-
Neuroticism/ Emotional Stability	anxiety, depression, negative affect	-	O

*Note*. + denotes positive associations; O denotes null associations;—denotes negative associations.

Despite the strengths of this review, including a broad range of methodologies and large, pooled samples, we note that we our analyses were largely reliant on self-reports of the two core construct gathered from convenience samples. While this is the case for much of the empirical literature on narcissism and self-esteem, it will be important to consider how these results might differ if tested in other relevant samples (e.g., forensic, corporate, clinical) and with other assessments approaches to narcissism and self-esteem (e.g., informant reports; interviews). Another limitation is our use of retrospective reports of developmental experiences, which are likely influenced by a myriad of other, contemporaneous factors. Furthermore, our data compilation and analyses should not be considered formal meta-analyses, as we did not seek any data outside of our own research labs. Finally, a related consideration is that we limited our analyses to narcissism and self-esteem as operationalized by the NPI and the RSES, respectively. The exclusive use of the NPI is likely more controversial, as there are active debates about the nature of narcissism as a construct and the best way to measure it [128]. However, the NPI yields empirical profiles that are strongly associated with expert ratings of grandiose narcissism and Narcissistic Personality Disorder [129], using an objective and quantitative approach to construct validation [23, 130].

We believe this to be among the broadest comparisons of the nomological networks of narcissism and self-esteem to date. Untangling these constructs is important, as it may have implications for interventions, and as such we advocate for measuring them concurrently. Nonetheless there are still many important questions to answer about these constructs, including (but not limited to) the potential for vacillations in self-esteem in response to ego-threat, the variance in self-esteem by domain, and the question of causality (i.e., do positive life events cause self-positivity, or does high self-positivity cause positive life events?). Thus, we hope this review will serve as a comprehensive resource for understanding the similarities and differences lie between narcissism and self-esteem, which appear to be trait-based, consistent across methodologies, and key to important mental health outcomes.
